# IEEE 802.11-Enabled Wake-Up Radio: Use Cases and Applications

**DOI:** 10.3390/s20010066

**Published:** 2019-12-21

**Authors:** Elena Lopez-Aguilera, Ilker Demirkol, Eduard Garcia-Villegas, Josep Paradells

**Affiliations:** 1 Department of Network Engineering, Universitat Politècnica de Catalunya, 08034 Barcelona, Spain; eduardg@entel.upc.edu (E.G.-V.); josep.paradells@entel.upc.edu (J.P.); 2 Department of Mining, Industrial and ICT Engineering, Universitat Politècnica de Catalunya, 08240 Manresa, Spain; ilker.demirkol@entel.upc.edu

**Keywords:** wake-up radio, IEEE 802.11, IEEE 802.11ba, internet of things, green networks

## Abstract

IEEE 802.11 is one of the most commonly used radio access technologies, being present in almost all handheld devices with networking capabilities. However, its energy-hungry communication modes are a challenge for the increased battery lifetime of such devices and are an obstacle for its use in battery-constrained devices such as the ones defined by many Internet of Things applications. Wake-up Radio (WuR) systems have appeared as a solution for increasing the energy efficiency of communication technologies by employing a secondary low-power radio interface, which is always in the active state and switches the primary transceiver (used for main data communication) from the energy-saving to the active operation mode. The high market penetration of IEEE 802.11 technology, together with the benefits that WuR systems can bring to this widespread technology, motivates this article’s focus on IEEE 802.11-based WuR solutions. More specifically, we elaborate on the feasibility of such IEEE 802.11-based WuR solutions, and introduce the latest standardization efforts in this IEEE 802.11-based WuR domain, IEEE 802.11ba, which is a forthcoming IEEE 802.11 amendment, discussing its main features and potential use cases. As a use case consisting of green Wi-Fi application, we provide a proof-of-concept smart plug system implemented by a WuR that is activated remotely using IEEE 802.11 devices, evaluate its monetary and energy savings, and compare it with commercially available smart plug solutions. Finally, we discuss novel applications beyond the wake-up functionality that IEEE 802.11-enabled WuR devices can offer using a secondary radio, as well as applications that have not yet been considered by IEEE 802.11ba. As a result, we argue that the IEEE 802.11-based WuR solution will support a wide range of devices and deployments, for both low-rate and low-power communications, as well as high-rate transmissions.

## 1. Introduction

IEEE 802.11 (Wi-Fi)-based Wireless Local Area Networks (WLAN), with more than 9.5 billion devices currently in use around the world, have experienced significant growth in the last decade. Widespread IEEE 802.11 is the preferred radio access technology for indoor wireless communications with the highest market penetration, being present in almost all consumer electronics devices with network capabilities (smartphones, tablets, notebooks, etc.). However, IEEE 802.11 technology has not yet been considered for building either home automation solutions or wireless sensor networks due to the high price of the chips and their high power consumption. Nevertheless, with mass production the price gap is disappearing, e.g., IEEE 802.11 Espressif chip ESP8266 costs $1, which is cheaper than IEEE 802.15.4 or Bluetooth Low Energy (BLE) solutions. On the other hand, IEEE 802.11 has been used in devices that are powered by the mains supply or by batteries recharged quite frequently, and power consumption still remains a concern for standby, transmission, and reception modes. For example, Espressif chip ESP32 [1] (evolved ESP8266) requires 190 mA when transmitting at 54 Mbps with a power of 16 dBm. Another challenge is the infeasibility of a small size battery to provide the required peak current, although this limitation can be overcome with the use of supercapacitors allowing the storage of necessary energy to cover power peaks. Nevertheless, the main issue for IEEE 802.11 solutions appears in reception, with a current consumption of 95–100 mA, being the reception instant unknown in advance and thus requiring longer time for the reception process. The receiver has to be continuously in the active state, as transmission can occur at any time, or it can enter the active state periodically if a duty cycle is defined. The last option has been the solution commonly adopted for battery powered devices, but at the cost of introducing delay in the transmission, synchronization overhead, and energy waste due to idle listening and the use of low-precision clocks. Moreover, considering only power peak is not fair when comparing IEEE 802.11 and IEEE 802.15.4 technologies. If we consider the energy per frame in transmission and in reception modes, IEEE 802.11 offers clear advantages over IEEE 802.15.4. One of the most power efficient IEEE 802.15.4 chips, TI CC2630 [2], consumes 73 μJ (with a power of 0 dBm) for the transmission of a 125 byte frame, and 70.8 μJ for its reception. For the IEEE 802.11 ESP32 chip, the energy per such frame is 10.55 μJ in transmission (with a power of 16 dBm and 54 Mbps) and 5.5 μJ in reception, thus, IEEE 802.11 offers an energy saving of one order of magnitude with respect to IEEE 802.15.4, and thereby is better suited for devices with a limited source of energy.

On the other hand, it is expected that the number of sensor nodes (devices able to collect sensory information and to communicate within a network) with non-cellular connections will exceed the number of consumer electronics devices in the near future, and that such sensor nodes will account for more than half of the Internet of Things (IoT) devices. With these devices being powered by batteries or energy harvesting, it is of key importance to use low-power equipment and further reduce energy consumption. For this reason, different initiatives are currently being developed within the IEEE 802.11 Work Group (WG) to link aforementioned two paradigms; one example is the IEEE 802.11ah standard, intended to define low-power and long-range WLAN communications, and a further step is expected from the IEEE 802.11 task group TGba, currently working on a Wake-up Radio (WuR) standard for IEEE 802.11 WLAN.

**Figure 1:**
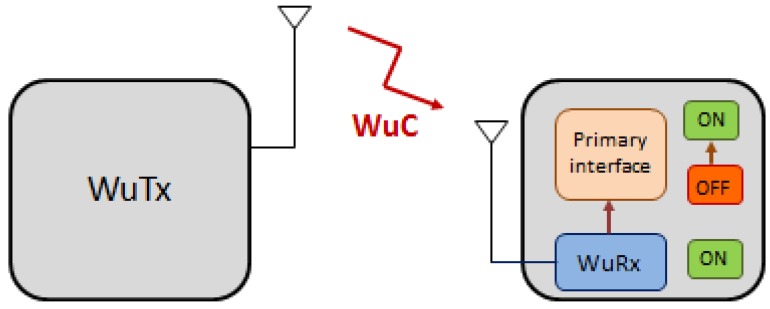
WuR (Wake-up Radio) system diagram.

Compared to the duty cycle solutions, where the receiver goes to a sleep state and wakes up periodically for the defined duty cycle, WuR systems achieve higher energy efficiency by employing two transceivers at the receiver: A primary transceiver for main data communication and a secondary receiver used to switch the primary transceiver from an energy-saving state to active operation (Figure 1). This secondary receiver, called the WuR receiver (WuRx), has very low-power consumption and, therefore, can be permanently active without a significant sacrifice in terms of battery life. The WuRx wakes up the primary radio upon reception of a WuR call (WuC) signal from a WuR transmitter (WuTx). Moreover, in addition to the energy saving use case, WuR solutions can be used for synchronization, or for notifying a device about a relevant event, thus alerting a receiver about the next transmission. Thus, due to the aforementioned reasons, the IEEE 802.11 TGba (working on the forthcoming IEEE 802.11ba specification) is focused on the WuR standardization employing IEEE 802.11 technology. The WuR solution to be adopted by TGba would result in a single integrated solution for both low-rate and efficient low-power communications, but also for high-rate traffic transmissions, thus making this specification applicable in a wide range of devices and deployments.

In this article, we study the feasibility and advantages of IEEE 802.11-based WuR solutions, which are built on widely adopted IEEE 802.11 technology, thus exploiting the benefits of WuR systems to achieve energy efficient communication. More specifically, we review the existing WuR systems, focusing on IEEE 802.11-based WuR solutions (Section 2), and show the forthcoming IEEE 802.11ba amendment, discussing potential cases of its use (Section 3). For the use case of a green Wi-Fi application, we show the feasibility of such WuR solutions through a proof-of-concept (PoC) smart plug implementation of an IEEE 802.11-based WuR using off-the-shelf devices, evaluating its monetary and energy savings, and comparing it with commercially available solutions (Section 4). Finally, potential future applications of IEEE 802.11-based WuR solutions are depicted, whilst also showing that a Wi-Fi WuR receiver can be used for novel purposes beyond the wake-up functionality, and thus, beyond the TGba focus (Section 5). Following this discussion, the article is concluded in Section 6.

## 2. Wake-Up Radio Systems So Far

In this section we provide a thorough review of the state of the art on WuR systems.

Most wake-up communication solutions focus on the use of radio frequency (RF) signals [3], although there are solutions built on acoustic [4] and Infra Red (IR) [5] technologies. WuR can be classified as active or passive based on whether they require an attached power source, addressable or non-addressable based on whether a specific node or group of nodes can be specified in the WuC, and configurable or non-configurable based on whether the WuRx settings can be modified, e.g., its address or the communication settings.

To reduce the power requirement of WuRx, a trivial approach is to simplify its circuit design. The first proof-of-concept for WuRx in the literature, in fact, only features a capacitor and a rectifying diode [6]. Albeit being non-configurable, this low-complexity solution is addressable, for which the WuTx transmits signals at specific frequencies simultaneously to activate an intended WuRx. Yet, this WuR system reaches a very limited operational range of only a few meters. A common method to achieve low-power WuRx is the use of envelope detection techniques to eliminate the power hungry local oscillators used in conventional radios. However, this creates a challenge for the development of high frequency WuRx and, hence, for high rate and low delay solutions.

Another common approach for efficient WuRx design is the use of hardware correlators. In that approach, a shift register stores the address demodulated from a WuC by an analog to digital conversion (mostly performed by envelope detectors). At each clock cycle, the register bits are shifted by adding the newly received bit. Then, the correlator circuit correlates the received bits to a pre-stored array of bits corresponding to a preamble and/or WuRx address. In case of a match, the wake-up interrupt is generated (the wake-up pin is asserted). Correlator circuits can consume only few μA when in idle state and around 10 μA when fully active and decoding the WuC address [7]. Hence, all correlator-based WuRx designs implicitly feature addressing capabilities. The addressable WuRx proposed in [8] is one of the lowest power solutions in the literature, consuming a mere 4.5 nW. To achieve such low power with an impressive −63.8 dBm sensitivity, its design also uses the envelope detector and correlator approach.

### IEEE 802.11-Based WuR Solutions

More specifically, there have been several studies focusing on the implementation of IEEE 802.11-based WuR systems in the literature. First, in [9], legacy IEEE 802.11 Beacon frames are used for generating a WuC. Upon energy detection over a threshold, the WuRx wakes up the main radio. IEEE 802.11 legacy transmitters can be used to generate the WuC, however, the system shows an important drawback, as the number of false positive detections is high, due to noise and interfering signals. Thus, the same authors incorporate in [10] an address pattern, in order to overcome this issue. This proposal alternates the transmission of long IEEE 802.11 legacy data frames and silence periods, to generate *on* and *off* periods respectively, and to code address patterns in a Non-Return-to-Zero (NRZ) manner. Nevertheless, the long silent periods make this proposal still prone to interference. In [11], a WuR is proposed where the legacy IEEE 802.11 frame lengths are used for address coding. In addition, this proposal provides various mechanisms to reduce the number of false positive detection and, thus, is more resilient to interference. Nevertheless, the consumption of the WuRx design is in the mW order, which is too high for a WuRx. On the other hand, following a different approach, in [12] a WuR system is proposed with the transmission of modified IEEE 802.11 Orthogonal Frequency Division Multiplexing (OFDM) symbols to build the WuC and address identifiers. The subcarriers that compose the OFDM signal are grouped in different sets, forcing some of them to zero, and building in this sense the address pattern in the frequency domain. This proposal has only been validated through simulation and its implementation of the WuC by employing legacy IEEE 802.11 transmitters is infeasible. In [13], a WuR system is presented by building the WuC by controlling the data pattern of OFDM subcarriers and producing an amplitude modulated signal composed of subsequent OFDM symbols. The IEEE 802.11ah OFDM symbol duration has been used for this purpose. Unfortunately, the complete system block diagram of IEEE 802.11 transmitters has not been taken into account, thus resulting in the system not being implementable by legacy IEEE 802.11 equipment. In [14], a WuR system is presented with the WuC being generated through a single IEEE 802.11 frame. It is modulated with an amplitude-based digital modulation employing the properties of the OFDM PHY. However, this proposal only provides evaluation through simulation. In [15], a WuR system solution is proposed, where the WuC consists in On-Off Keying (OOK) modulated data incorporated into a single IEEE 802.11 frame, and with a WuRx consumption of 95 μW. Although the complete system implementation is presented, legacy IEEE 802.11 transmitters need a firmware update for WuC development. In [16] we presented a WuR system comprising of a low-power receiver (10 μW). Unlike in previous references, the transmitter can be implemented on any IEEE 802.11-enabled device without requiring any firmware or hardware modification, including smartphones. The WuC is modulated using OOK, where on periods are generated by the transmission of subsequent legacy IEEE 802.11 broadcast frames of minimum length, and off periods are achieved by means of a silence period. After significant scientific community interest in this topic, standardization efforts were started within IEEE. Specifically, IEEE 802.11 WG initiated the task group TGba in December 2016, aiming at developing a new IEEE 802.11 amendment for WuR operation [17,18]. In Section 3 we present the forthcoming IEEE 802.11ba amendment.

## 3. IEEE 802.11ba Amendment

Since December 2016, IEEE 802.11 WG TGba has worked on the IEEE 802.11 amendment development for WuR operation, i.e., the latest standardization effort in the IEEE 802.11-based WuR domain. Earlier, in July 2015, a new Topic Interest Group (TIG) on Long-Range Low-Power (LRLP) operation for IoT was started, which aimed to bring some of the new IEEE 802.11ah features (targeting 868/915 MHz bands) to the 2.4 GHz band while keeping compatibility with mainstream IEEE 802.11 devices in that band. In May 2016, the TIG agreed to focus on the low-power feature (leaving aside the long range topic), thus creating the Low-Power WuR Study Group (LP-WuR SG), and dissolving the LRLP TIG. From May to November 2016 LP-WuR SG worked to meet the requirements needed to become a task group within the IEEE 802.11 WG, in charge of producing the new IEEE 802.11ba amendment. The new task group TGba, approved in December 2016, is currently working on the draft document, defining PHY and MAC layer specifications to enable the operation of a WuR system [17].

### 3.1. Usage Models

**Figure 2:**
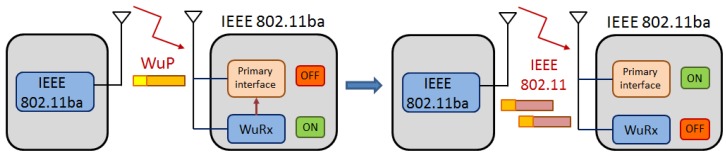
IEEE 802.11ba usage case basic operation.

In order to foresee the requirements that future IEEE 802.11ba technology is expected to meet, engineers try to imagine different scenarios where the use of IEEE 802.11-based WuR will bring evident benefits. In this way, TGba first defined its target usage models [19]. The challenges identified in these usage models are then translated into functional requirements to guarantee that the resulting specification makes the new technology capable of enabling them. Most usage models discussed within the TGba are built on the following basic operation: (i) The primary radio (and possibly other energy-hungry systems) of an IEEE 802.11ba-capable low-power device is put to sleep in order to save energy; (ii) when communication with the sleeping device is required, a WuR packet (WuP) is sent in order to generate the WuC to wake up the device’s primary radio; and (iii) normal IEEE 802.11 communication happens through the primary radio (Figure 2). This basic operation poses three requirements that are common to all studied cases. First, since primary radios are responsible for sending WuPs, coexistence of these new frames with legacy IEEE 802.11 transmissions must be enabled. Second, the range of WuR communication should be the same as the one of the primary radio so that normal IEEE 802.11 operation is possible after the wake-up. Third, WuR-capable devices must be uniquely identified so that the WuP has effect only on the intended recipient. However, some of the studied usage models will require additional functionalities, as discussed below.

*Smart home*: A domestic environment, where one access point (AP) serves stations (STA) without power limitations (e.g., TV, PC, household appliances) sharing space with tens of low-power battery-driven sensors/actuators of smart home applications (e.g., smart-metering, assisted-living, security/safety, home automation). In this scenario, WuP transmission copes with intensive use of the Wi-Fi channel (e.g., due to video streaming, gaming).

*Warehouse*: A large indoor space served by multiple APs. Each container/box or each shelf is equipped with sensors that report, on-demand, the location or state of the stored goods to a central server or to the warehouse workers’ handheld devices. In this scenario, each AP should support several hundreds of IEEE 802.11ba-capable devices.

*Cattle farm*: Outdoor scenarios in which the livestock carries different sensors. The farmers are equipped with mobile phones which work as APs serving the mobile sensors. Farmers use the phones to track the state (e.g., body temperature, location) of each animal independently. Upon request, those mobile phones will wake up the device mounted on the animal before querying its state through the main radio. However, mobile phones will also benefit from energy savings; thus, this scenario introduces the possibility that an AP is equipped with a WuRx so that it can switch to sleep mode while allowing the delivery of event-triggered reports from the sensors to the sleeping AP through an AP-destined WuP.

*Wearable devices*: In this scenario, personal or body area networks in fitness/health applications are built by connecting different wearable devices (e.g., smartwatch, chest strap) to a smartphone via Wi-Fi. Similar to the cattle farm case, this scenario also considers bi-directional WuP transmission whereby the phone could poll periodically different wearable devices and, in the opposite direction, a heart-rate sensor, for example, wakes up the smartphone to trigger an automatic emergency call upon detection of a heart failure.

*Moving goods*: Freighted containers or parcels, equipped with IEEE 802.11ba-capable sensors, are located and tracked from a central server. While in motion or upon arrival to a transfer station/warehouse, local APs wake up those sensors to record their current location. Note that, in this case, a unique identifier within the scope of the AP is not enough as the identifier must be unique in a more global context.

*Intelligent Transportation System (ITS)*: Vulnerable road users (e.g., pedestrians or cyclists) need power-efficient communication devices in order to facilitate its integration within an ITS. In an example of such an application, when a pedestrian approaches a dangerous intersection or moving vehicle, the infrastructure or the vehicle itself sends WuP so that the pedestrian-carried device turns on automotive safety radio operation. This case requires support for multicast/broadcast transmission and operation outside the context of the Basic Service Set (as defined in IEEE 802.11p [20]).

### 3.2. PHY and MAC Characteristics

**Figure 3:**
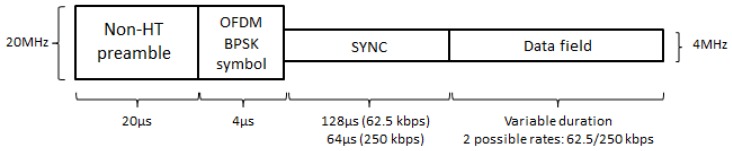
IEEE 802.11ba WuR 20 MHz PPDU (PHY Protocol Data Unit).

In the PHY layer being specified by the TGba, the 20 MHz non-High Throughput (non-HT) preamble will be used for any WuR PHY Protocol Data Unit (PPDU), immediately followed by a 20 MHz OFDM symbol with Binary Phase Shift Keying (BPSK) modulation of 4 μs duration, the Synchronization (SYNC), and the Data fields (Figure 3). The employment of non-HT preamble allows WuR frame transmissions to be sensed by any legacy IEEE 802.11a/g/n/ac station, building the 20 MHz non-HT preamble and the BPSK OFDM symbol together with the 20 MHz WuR PPDU preamble. On the other hand, SYNC and Data fields consist in the narrowband portion of the WuR PPDU, as the number of OFDM subcarriers employed is reduced to 13 subcarriers spaced 312.5 kHz each, i.e., SYNC and Data fields occupy 4 MHz of bandwidth instead of 20 MHz, thus leading to a simplified lower power receiver while still maintaining a suitable Signal to Interference and Noise Ratio (SINR). The aforementioned described PPDU is referred to as WuR PPDU with a 20 MHz channel bandwidth.

**Figure 4:**
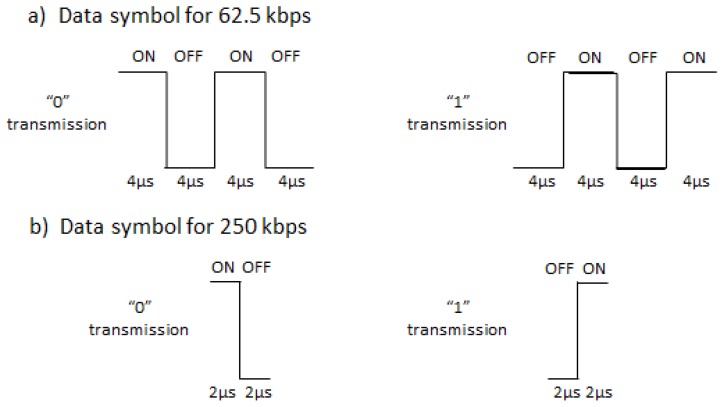
IEEE 802.11ba WuR symbol structure: (**a**) Data symbol for 62.5 kbps, (**b**) Data symbol for 250 kbps.

Only two rates are supported to achieve a simplified receiver: A high rate of 250 kbps (assuming a 1 bit per legacy IEEE 802.11 symbol of 4 μs), and a low rate of 62.5 kbps (as a more robust communication mode for noisier environments). SYNC field consists of being in the narrowband preamble dealing with synchronization, frame detection, and rate indication functionalities. Two different SYNC durations (64 μs or 128 μs) are employed depending on the bit rate used in the Data field (the high-rate of 250 kbps or the low-rate of 62.5 kbps, respectively), with SYNC field bit sequences being chosen for a reduced Frame Error Rate (FER). Moreover, Multi-Carrier (MC)-OOK modulation with Manchester coding is being employed for the Data field symbols, which provides on and off periods for both one and zero bit values, thus avoiding continuous large off periods leading to false idle medium detection by surrounding stations. Symbol’s duration depends on the bit rate, and its structure on whether a one or a zero bit value is being transmitted. In this way, for 62.5 kbps, a 16 μs long symbol is employed, with four differentiated sub-parts of 4 μs each alternating on and off periods, following a different schedule for transmissions of one and zero bits (Figure 4a). On the other hand, for 250 kbps, symbol duration is 4 μs with two sub-parts of 2 μs each, also alternating the on and off periods differently for transmissions of one and zero bits (Figure 4b).

**Figure 5:**
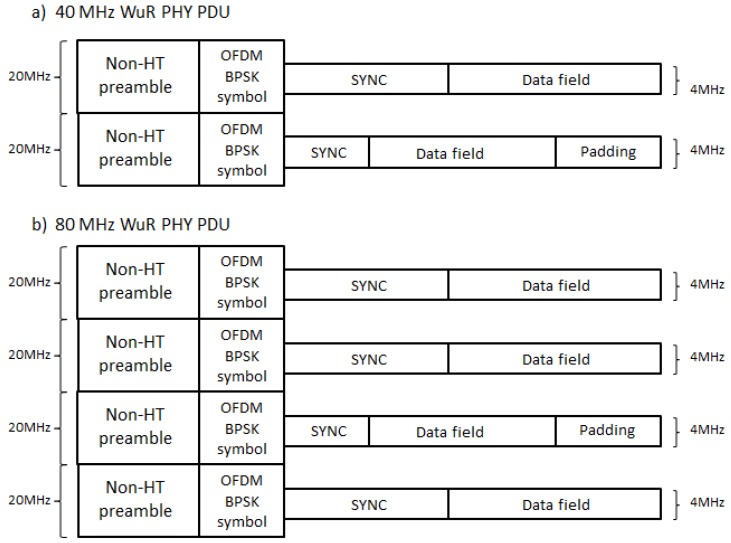
IEEE 802.11ba 40 MHz and 80 MHz WuR PPDU: (**a**) 40 MHz WuR PPDU, (**b**) 80 MHz WuR PPDU.

TGba PHY provides optional support for 40 MHz and 80 MHz channel bandwidth. In this case, WuR PPDUs with 40 MHz and 80 MHz channel bandwidth, respectively, are transmitted using Frequency Division Multiple Access (FDMA), where the 20 MHz WuR PPDU preamble is duplicated in each 20 MHz sub-channel, thus allowing WuR frames transmissions to be sensed by any IEEE 802.11n/ac station in both primary and secondary channels. Subsequently, the 4 MHz WuR signal centered in the 20 MHz sub-channel is transmitted. Moreover, transmissions in separated sub-channels can use different bit rates (the high-rate of 250 kbps or the low-rate of 62.5 kbps), thus, different SYNC field sequences (with corresponding distinct duration) and data field durations may be applied. As the WuR PPDU in each 20 MHz sub-channel needs to have an equal duration of transmission, the padding may be used to ensure this condition (Figure 5).

The MAC layer defines the WuR mode to where stations are allowed to enter after WuR mode negotiation and explicit signaling. Stations in the WuR mode have their primary radio in sleep state and the secondary radio actively listening for WuP. Once the WuP is received, the station changes the state of the primary radio from sleep to awake, replies to the sender, and can turn off the secondary radio. Additionally, stations may support a duty cycle mode for WuR mode operation, with active and inactive periods also in the secondary radio, thus allowing higher power savings. Enhanced Distributed Channel Access (EDCA) with any of its four access categories is used as a channel access mechanism to send WuP, thus allowing coexistence with legacy IEEE 802.11 frames. Unlike legacy MAC, if transmission fails, it is retried without increasing the contention window value, which avoids additional delays in the WuP transmission, and thus in WuR operation.

**Figure 6:**
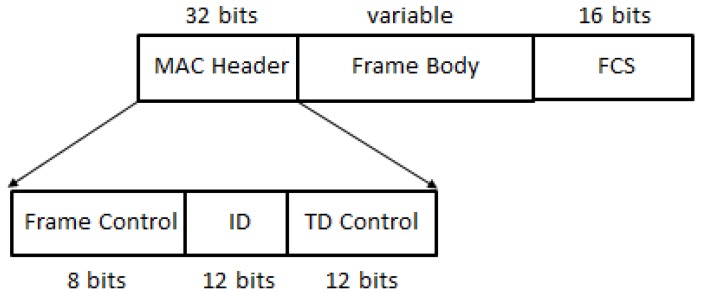
IEEE 802.11ba MAC frame structure.

The MAC frame structure of WuR frames is depicted in Figure 6 and contains a simplified MAC header field (composed by Frame Control, ID, and Type Dependent (TD) Control fields), an optional frame body of variable length and a Frame Checksum Field (FCS), as for legacy IEEE 802.11 frames. The Frame Control field inside the MAC header identifies the type of frame (as for legacy IEEE 802.11 frames), but limiting the frame types to Beacon, WuP, Discovery (to assist stations in WuR mode for WuR AP discovery through selected discovery channel scanning), and Vendor Specific frames. The ID field included in the MAC header does not correspond to the MAC address as in IEEE 802.11 legacy frames, but to an identifier that depends on the type of WuR frame. Thus, it can either identify the transmitting AP, or an individual station or a group of stations to which the WuR frame is addressed. The TD Control field contains information based on the specific WuR frame type.

## 4. A Proof-Of-Concept IEEE 802.11-Based WuR Implementation

In this section, we illustrate how IEEE 802.11 can be used to develop a WuR system and provide a real use case consisting of a green Wi-Fi application that is built on this IEEE 802.11-based WuR solution. For this, we first detail the WuR system, the preliminary version of which was presented in [16]. This WuR system consists of a low-power WuRx and a WuTx that can be implemented on any IEEE 802.11-enabled device, without any firmware or hardware modification. It provides a PoC IEEE 802.11-based WuR implementation using IEEE 802.11 off-the-shelf devices, as detailed in this section. It also allows us to show the energy and monetary cost savings that can be offered by an IEEE 802.11-enabled WuR system in this exemplary green Wi-Fi application. In this use case, Wi-Fi APs are moved to a power saving state when not in use, and woken up by a Wi-Fi STA upon request.

### 4.1. IEEE 802.11 as WuR Technology

**Figure 7:**
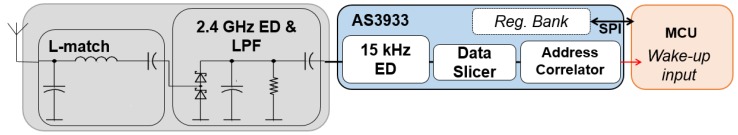
Proposed IEEE 802.11 WuRx (WuR receiver) implementation.

The system consists of a standard IEEE 802.11-capable device, which has the role of the WuTx, and a low-power WuRx. The WuRx, as depicted in Figure 7, is built by preceding the AS3933 [21] integrated circuit, with a 2.4 GHz antenna and the corresponding impedance matching stage. The AS3933 WuRx chip features a kHz-level high-performance envelope detector and an address correlator. The signal received is down-converted from the GHz to the kHz level, after impedance matching, by employing an envelope detector (ED) and a Low-Pass Filter (LPF). One inductance and two capacitors build the L-network that matches the 50 Ω output impedance of the antenna to the input impedance of the Schottky diode. Next, the AS3933 extracts the node address modulated in the WuP using an internal envelope detector. The data slicer block included in the AS3933 allows data delimitation according to the bit rate of the WuR system. An Interrupt ReQuest signal is generated to wake up the device from its sleep mode, upon detection of the device’s pre-configured address by the address correlator block in the AS3933.

**Figure 8:**
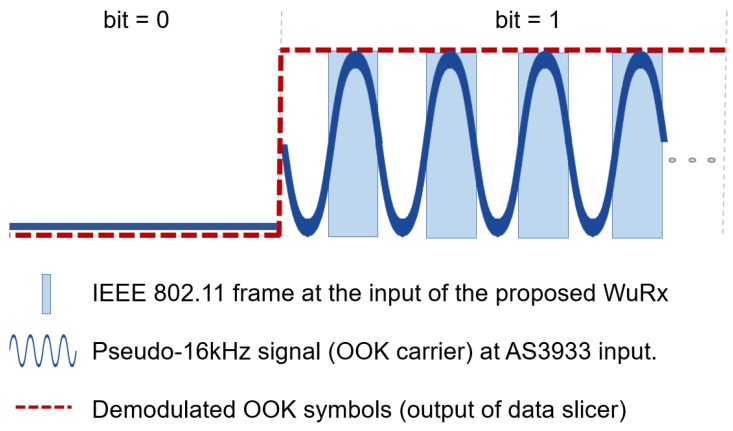
WuP (WuR packet) in proposed WuR implementation.

The WuRx presented listens for a kHz-level signal modulated in the 2.4 GHz band according to an OOK modulation. The proposed system follows the so-called SubCarrier Modulation (SCM) procedure [7], which is shown in Figure 8. It combines the transmission of a regular IEEE 802.11 frame bursts (ones) and silence periods (zeroes) to emulate a 16 kHz carrier. It is employed to OOK-modulate the WuP, which contains a preamble and an address field. Broadcast empty data frames are used for the transmission of regular bursts of standard IEEE 802.11 frames, since they do not require an acknowledged two-way exchange and can be sent with a minimum separation of a DCF inter-frame space (DIFS) time. Any device equipped with a Wi-Fi compatible device can generate this kind of signal only with software modifications. For this PoC, we use a desktop PC running Linux with a Qualcomm-Atheros IEEE 802.11g card supported by the open source MadWifi driver. We programmed a simple user-level application to generate the frame bursts (i.e., the emulated low-rate OOK signal for WuP generation). For a single wake-up event, a total wake-up latency of 45.87 ms was measured. We refer the reader to reference [16] for a detailed evaluation.

**Figure 9:**
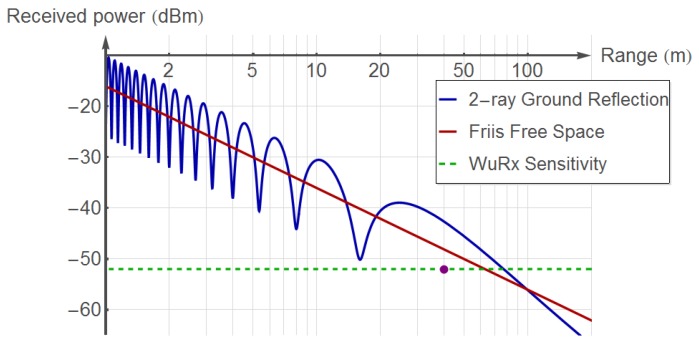
Operational ranges corresponding to the two theoretical propagation models for the WuRx sensitivity measured of −52 dBm. The red dot shows the maximum distance observed in the experimental measurements.

In our implementation, the WuRx featured a sensitivity of −52 dBm. Following the approach in [22], a led on the WuRx board was turned on by the MCU upon WuP reception, being the minimum power level measured to −52 dBm, while still correctly detecting the WuP. This sensitivity was translated into an effective range of near 40 m (in highly interfered outdoor scenario) when the wireless card was transmitting at +18 dBm with +2 dB of antenna gain. This analysis has been done experimentally in an outdoor highly interfered campus environment and in an interference-free indoor scenario using a test setup with WuTx and WuRx placed at a height of 1 m. The WuTx transmits 10 WuPs per second (up to a maximum of 100 WuPs), and the WuRx is progressively displaced away from the WuTx in steps of 2 m. Each measurement is averaged over 5 repetitions. No WuP is detected beyond 40 m [16]. Average delays of 2.3 s for the outdoor scenario have been measured, which are acceptable values for many applications, thus showing the robustness of the system in highly interfered scenarios. To compare this range with the theoretical limits, we simulate two well-known propagation models and depict the results in Figure 9. As seen in the figure, the operational range for the 2-ray Ground Reflection and Friis Free Space propagation models for the given sensitivity is 20 m and 35 m longer, respectively, than that of our WuRx prototype. Such deviations are reasonable due to the losses in the hardware implementation. The WuRx achieves a measured, remarkably low power consumption of 10.8 μW in sleep mode and of 24 μW during WuP reception and decoding [16]. These values include the power required by the AS3933 and the low-power MCU TI MSP430F2350 [23], which has been used in the WuRx board. This MCU requires 0.3 μW in its lowest power mode LPM4. This means a battery lifetime of between 3 and 7 years, depending on WuP arrival frequency and considering a common CR2032 battery with 225 mAh of capacity.

### 4.2. Proof-Of-Concept: Wi-Fi Controlled Smart Plug

By using the IEEE 802.11-based WuR system described in the previous section, we developed a smart plug, which deals with green Wi-Fi AP operation and can be controlled by any off-the-shelf IEEE 802.11 device. Two versions of the switch were designed: (i) The smart plug is powered directly from the mains voltage, for example by simply plugging it to a wall-mount plug; and (ii) A smart DC switch, which uses a DC voltage input, to be used, for example, between the power adapter and the electronic device.

The first case (AC solution) can be considered as a universal plug solution since it will allow the connection of many of the most common appliances, supporting a higher switching voltage/current than the DC option. However, the AC solution still requires an AC/DC conversion to feed the WuRx (3.3 V) and requires components that are slightly more expensive. The DC solution is powered with 12 V provided by the power adapter of the electronic device (i.e., the Wi-Fi access point in this case). Then, it is suitable for the specific case of the selected access point but also for all the DC devices that are in the electrical ranges of the designed components. The design of both approaches face different trade-offs, for example the choice of a mechanical relay or a solid-state relay. The latter has a longer life and switches faster while the mechanical relay (latching type), on the other hand, shows a peak of consumption only during switching time but wastes exactly 0 W during on and off periods, while solid-state counterparts cannot provide a perfectly open circuit and present small current leakage both in the on and off state.

**Figure 10:**
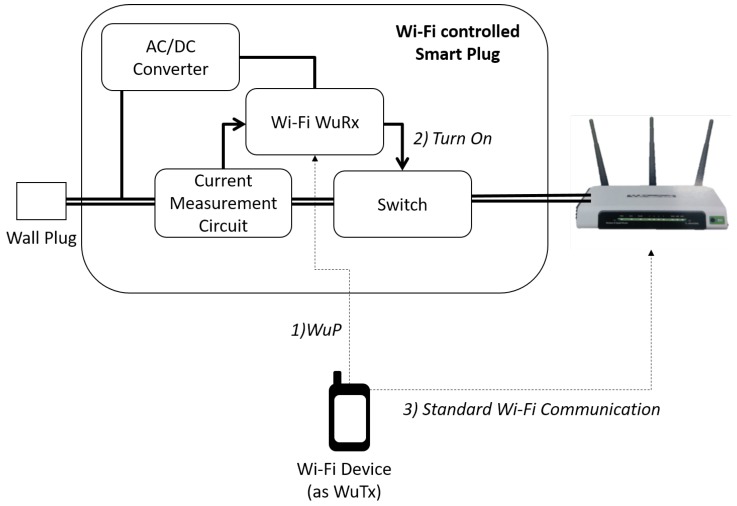
Wi-Fi controlled smart plug design.

The architectural design of our smart plug is given in Figure 10. The current measurement circuit is used to differentiate between the operational and the standby modes by comparing the actual current consumption to a reference value. This allows the plug to detect periods of low or no activity and automatically switch off the connected device. Although, for this study, we provided a fixed threshold (if measured current is below the threshold, state changes to off), and more generic solutions can be devised, for example, by registering the standby and active modes of an electronic device through user interaction. A 3.3 V power converter has also been designed with a non-isolated capacitive power supply to feed the WuRx.

In our PoC, we targeted a green Wi-Fi AP application, where an AP was turned off automatically if there was no station associated to it, and the same AP could be turned on remotely by a Wi-Fi STA by waking the smart plug up. For this, we used a commercial Wi-Fi AP, TP-Link WR1043ND, the power consumption of which was experimentally measured with a power analyzer (Agilent Technologies N6705A) in different states to configure the threshold for the current measurement circuit: (i) The AP is on and serving zero STA, (ii) the AP is on and serving one STA at least. It was found that, when the AP was on, the measured current consumed by the AP when serving zero or one STA was very similar (230 mA–250 mA vs. 230 mA–260 mA). This is expected since the AP is consuming a considerable power for listening the channel and responding to probe messages. In consequence, the current measurement circuit (cf. Figure 10) could not clearly distinguish the cases where the AP could be switched off to save energy (i.e., no STAs associated). In this case, some kind of action is needed from the AP to trigger a transition to the off state. Hence, we programmed the AP, running a Linux-based operating system, to execute the poweroff command, whenever there was no STA associated. When this command was executed, the measured current consumption was lowered to 160 mA–170 mA range, which allowed us to set a clear threshold to identify power saving opportunities.

After comparing the performance of different electrical components and their influence on the performance of the final solution (i.e., measuring their contribution to the energy consumption in on and off states of the switch), as well as their price, we developed two versions of the smart plug, namely DC and AC solutions, as discussed above. In both cases, we used low-cost off-the-shelf components, resulting in a device with an estimated cost between $7 and $10.

A first set of experiments were conducted to validate the correct operation of the WuRx shown in [16], that is, measure range, delay, and power consumed by the Wi-Fi WuRx element (see Figure 10). Then we experimentally quantified the power saving that could be achieved with this solution by measuring the power consumption in different states. In the operational state of the AP, the total measured power consumption was 4 W without the smart plug solution, but was increased to 4.3 W and 4.5 W with the DC and AC smart plug solutions, respectively. However, when there were no STAs associated, the AP was automatically turned off, as discussed in the previous paragraphs, reducing the measured power consumption to 0.5 W (DC) and 1 W (AC). Hence, compared to the most common scenario where APs remain always active regardless of their utilization, WuR-controlled AP solution reduced the power consumption by 87.5% (DC) and 75% (AC).

In the common use case of office WLANs, our developed WuR PoC solution allows the APs to be put to this energy saving mode most of the time, e.g., between 18:00 and 08:00. In a campus scenario, this mechanism translates to savings of around $6500 per year for an Extended Service Set (ESS) of 2000 APs, corresponding to the utilization of the WLAN measured in a large university campus [24]. Table 1 summarizes the performance of the two smart plug solutions, as well as the energy and monetary savings achieved in different scenarios.

The numbers shown in Table 1 correspond to the savings achieved when the smart plug is used with the lightweight TP-Link WR1043ND Wi-Fi AP. However, the developed smart plug solution supports appliances of up to 4 kW. For appliances that require such high power, this smart plug solution can achieve even more significant savings.

There are commercial smart plug solutions employing Wi-Fi communication and other radio frequency technologies such as Bluetooth, Zigbee, or GSM. In this regard, the Wemo Smart switch from Belkin [25] employs Wi-Fi and supports up to 1.8 kW appliances with a maximum power consumption of 1.5 W. The iSocket smart switch [26] uses GSM, supports up to 4 kW, and consumes 1 W. Both solutions can be remotely controlled from a smartphone device. The Valta Starter Kit [27] works with the 921 MHz frequency band, supports up to 1.8 kW appliances, and consumes up to 1.1 W. The Loxone Smart Socket [28] operates within the 868 MHz band and allows up to 4 kW with a power consumption of 0.5 W. However, the last two devices cannot be controlled from a smartphone, requiring an additional hardware for monitoring and configuration, which brings the important drawback of additional power consumption. In comparison to aforementioned commercial devices, our developed smart plug solution can be remotely controlled by smartphones employing Wi-Fi, consumes less than 1 W, and can control appliances of up to 4 kW of power consumption, thus showing that it is aligned with commercial devices. In addition, it consists of, in the first solution, a current (power) threshold to turn off the appliance automatically. Moreover, power consumption can be further reduced by removing the current measurement circuit and replacing the AC/DC converter of Figure 10 by a battery, as our smart plug incorporates a WuR system that allows one to considerably reduce power consumption ( Section 4.1). Finally, our smart plug design provides a lower-cost solution since it does not require a fully functional IEEE 802.11 chipset nor a MCU running a complete TCP/IP stack.

## 5. Beyond Wake-Up Functionality

**Figure 11:**
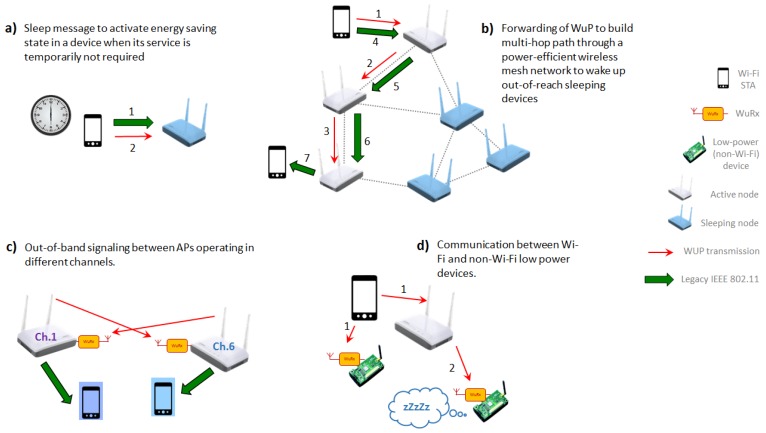
Potential use cases for a WuRx radio beyond of wake-up functionality: (**a**) Sleep message to activate energy saving state, (**b**) WuP forwarding, (**c**) Out-of-band signaling between APs, (**d**) Communications between Wi-Fi and non-Wi-Fi devices.

The presence of the secondary low-power radio interface in IEEE 802.11-based WuR solutions enables a wide range of new future applications beyond the wake-up functionality and the scope of TGba. Some of them are depicted in Figure 11 and are discussed below in this section.

Maybe the most obvious next step could be the use of a second type of packet other than the WuP, intended to switch battery-driven devices or the AP itself to an energy-saving state when required (e.g., out of office hours in an enterprise network), to achieve energy savings, reduced interference, or increased security (see Figure 11a). Another application not yet considered by TGba is WuP forwarding, which would allow the transmission of a WuP to devices out of the sender’s range, through a multi-hop path (see Figure 11b), where the range of WuR communication is not the same as the one of the primary radio.

The secondary radio could also be used to receive out-of-band signaling in a multi-AP environment, where neighboring APs operate in non-overlapping channels to avoid interference (see Figure 11c). In such scenarios, both APs and non-AP STAs often scan other channels to discover neighboring APs. With a secondary radio used for that purpose, those STAs’ primary radios will be able to keep operating in their home channel without any loss of connectivity. For that application, WuPs would include the initial configuration information such as channel number, bandwidth, Extended Service Set Identifier (ESSID), etc. In another exemplary embodiment of such functionality, APs could send other relevant signaling to neighboring APs without changing the frequency of the primary radio, for example, WuPs could include synchronization information when the intended receivers operate as anchors in time-measurement-based positioning (cf. IEEE 802.11az).

Moreover, IEEE 802.11ba implies the design of a low-power radio capable of receiving messages from IEEE 802.11-compatible devices, but this low-power radio could be mounted in any kind of device, which does not necessarily have an IEEE 802.11-based primary radio (see Figure 11d). This would enable communication between Wi-Fi devices and any non-Wi-Fi device. Nowadays, most (if not all) handheld devices with networking capabilities are equipped with an IEEE 802.11 interface. However, up until now and despite Wi-Fi HaLow (IEEE 802.11ah) [29], IEEE 802.11 has not shown a significant presence in the IoT market, where other technologies are well established (e.g., ZigBee/IEEE 802.15.4e, BLE, and different proprietary technologies). Therefore, it is critical to provide a means of communication between these two worlds: Personal communication devices and IoT. We believe TGba’s low power and cheap radio could be used for that matter. For example, a low-power sensor/actuator with a primary radio different from IEEE 802.11 could be equipped with an IEEE 802.11ba-like WuRx for wake-up purposes, but also to receive short messages or simple control information (e.g., for initial configuration, neighbor discovery, interference avoidance) from a Wi-Fi device. Furthermore, WuRx could be installed in appliances with no primary radio at all. In those cases, the WuRx would allow a unidirectional communication whereby a Wi-Fi device, acting as a remote control, can send simple commands to actuators (e.g., garage door opener, window blinds, thermostat). Then, (non-IEEE 802.11) recipients of the WuP react accordingly, not being required to provide any feedback to the sender.

## 6. Conclusions

In this article we analyzed the eligibility of using IEEE 802.11-based WuR solutions. First, we provided a review of WuR systems, and present the forthcoming IEEE 802.11ba amendment, which aimed at developing the new IEEE 802.11 specification for WuR standardization. We showed and discussed the potential use cases, and provided a proof-of-concept IEEE 802.11-based WuR implementation to illustrate the use case consisting in a green Wi-Fi application. A comparison with commercially available solutions was shown, including its monetary and energy savings. Thus, we presented a complete operative WuR system using IEEE 802.11 off-the-shelf devices portraying its feasibility and significant advantages. Finally, we highlighted the possibility of using the secondary radio interface of IEEE 802.11-enabled WuR devices for new applications beyond the wake-up functionality, thus depicting future applications not yet considered by IEEE 802.11ba.

## Figures and Tables

**Table 1:** Smart plug performance (P. cons.: Power consumption, P. sav: Power saving).

	Offices Building	University Campus	Household
**Active hours**	08:00–18:00	08:00–22:00	18:00–00:00
**Number of APs**	100	2000	1
**P. cons. w/o plug**	(4 W × 24 h × 100)	(4 W × 24 h × 2000)	(4 W × 24 h × 1)
**P. cons. w/ DC sol.**	(4.3 W × 10 h + 0.5 W × 14 h) × 100	(4.3 W × 14 h + 0.5 W × 10 h) × 2000	(4.3 W × 6 h + 0.5 Wx18 h) × 1
**P. sav. w/ DC sol.**	1679 kWh × year	22484 kWh × year	22.3 kWh × year
**P. cons. w/ AC sol.**	(4.5 W × 10 h + 1.0 W × 14 h) × 100	(4.5 W × 14 h + 1.0 W × 10 h) x 2000	(4.5 W × 6 h + 1.0 W × 18 h) × 1
**P. sav. w/ AC sol.**	1350 kWh × year	16790 kWh × year	18.6 kWh × year
**Price of electricity**	$0.29 kWh	$0.29 kWh	$0.29 kWh
**$ saving w/ DC sol.**	$487 × year	$6520 × year	$6.5 × year
**$ saving w/ AC sol.**	$392 × year	$4869 × year	$5.4 × year
